# Non-surgical treatment of pain associated with posterior tibial tendon dysfunction: study protocol for a randomised clinical trial

**DOI:** 10.1186/s13047-015-0095-4

**Published:** 2015-08-14

**Authors:** Angela Blasimann, Patric Eichelberger, Yvonne Brülhart, Isam El-Masri, Gerhard Flückiger, Lars Frauchiger, Martin Huber, Martin Weber, Fabian G. Krause, Heiner Baur

**Affiliations:** Bern University of Applied Sciences, Health, Physiotherapy, Murtenstrasse 10, 3008 Bern, Switzerland; Salem-Spital, Foot Surgery, Schänzlistrasse 39, 3013 Bern, Switzerland; Sonnenhofspital, Foot Surgery, Buchserstrasse 30, 3006 Bern, Switzerland; Spital STS AG, Orthopaedics & Traumatology, Krankenhausstrasse 12, 3600 Thun, Switzerland; Outpatient Clinic for Foot Surgery Bern, Schänzlistrasse 33, 3013 Bern, Switzerland; Siloah, Clinic for Orthopaedics and Traumatology, Worbstrasse 316, 3073 Gümligen bei Bern, Switzerland; Department of Orthopaedic Surgery, University of Bern, Inselspital, Freiburgstrasse, 3010 Bern, Switzerland

**Keywords:** Exercise therapy, Flatfeet, Flat foot, Foot orthoses, Gait, Pes planus, Resistance training

## Abstract

**Background:**

Symptoms associated with pes planovalgus or flatfeet occur frequently, even though some people with a flatfoot deformity remain asymptomatic. Pes planovalgus is proposed to be associated with foot/ankle pain and poor function. Concurrently, the multifactorial weakness of the tibialis posterior muscle and its tendon can lead to a flattening of the longitudinal arch of the foot. Those affected can experience functional impairment and pain. Less severe cases at an early stage are eligible for non-surgical treatment and foot orthoses are considered to be the first line approach. Furthermore, strengthening of arch and ankle stabilising muscles are thought to contribute to active compensation of the deformity leading to stress relief of soft tissue structures. There is only limited evidence concerning the numerous therapy approaches, and so far, no data are available showing functional benefits that accompany these interventions.

**Methods:**

After clinical diagnosis and clarification of inclusion criteria (e.g., age 40–70, current complaint of foot and ankle pain more than three months, posterior tibial tendon dysfunction stage I & II, longitudinal arch flattening verified by radiography), sixty participants with posterior tibial tendon dysfunction associated complaints will be included in the study and will be randomly assigned to one of three different intervention groups: (i) foot orthoses only (FOO), (ii) foot orthoses and eccentric exercise (FOE), or (iii) sham foot orthoses only (FOS). Participants in the FOO and FOE groups will be allocated individualised foot orthoses, the latter combined with eccentric exercise for ankle stabilisation and strengthening of the tibialis posterior muscle. Participants in the FOS group will be allocated sham foot orthoses only. During the intervention period of 12 weeks, all participants will be encouraged to follow an educational program for dosed foot load management (e.g., to stop activity if they experience increasing pain). Functional impairment will be evaluated pre- and post-intervention by the Foot Function Index. Further outcome measures include the Pain Disability Index, Visual Analogue Scale for pain, SF-12, kinematic data from 3D-movement analysis and neuromuscular activity during level and downstairs walking. Measuring outcomes pre- and post-intervention will allow the calculation of intervention effects by 3×3 Analysis of Variance (ANOVA) with repeated measures.

**Discussion:**

The purpose of this randomised trial is to evaluate the therapeutic benefit of three different non-surgical treatment regimens in participants with posterior tibial tendon dysfunction and accompanying pes planovalgus. Furthermore, the analysis of changes in gait mechanics and neuromuscular control will contribute to an enhanced understanding of functional changes and eventually optimise conservative management strategies for these patients.

**Trial registration:**

ClinicalTrials.gov Protocol Registration System: ClinicalTrials.gov ID NCT01839669

## Background

The foot deformity pes planovalgus can cause symptoms or complaints, and may lead to dysfunction and functional limitation [1–3]. It can be an acquired entity where weakness of the plantarflexors and invertors of the foot (mainly the tibialis posterior muscle) cause a flexible or rigid flatfoot deformity [4, 5].

The posterior tibial tendon/muscle primarily serves as a dynamic stabiliser of the foot’s medial longitudinal arch [6]. Excessive stress can lead to tendinopathy of the posterior tibial tendon. This leads to posterior tibial tendon dysfunction (PTTD). Therefore, PTTD is most often accompanied by pes planovalgus foot deformity. Because of the mutual prevalence of both aspects, it is not clear if the muscle-tendon dysfunction is a result of the foot deformity [7, 8], or if it is causative for foot deformation [9, 10]. There is consensus that PTTD and pes planovalgus are closely connected [11–15].

Patients with PTTD and concomitant pes planovalgus report pain around the medial malleolus in the tibialis posterior muscle and its tendon. As the condition progresses, they can exhibit plantar hyperkeratosis in the talus area and lateral impingement at the fibula-calcaneal transition [2, 14, 15].

PTTD can be divided into four stages according to Johnson & Strom [2]. Stage I and II comprise a still flexible foot structure, which is considered to be eligible for non-surgical treatment. Stage III and IV contain already rigid longitudinal arch flattening with a tear of the spring ligament complex and talar valgus tilt in the ankle mortise [16]. Here, active compensation is not able to take place [17, 2].

As long as PTTD and the foot deformity demonstrate no symptoms, treatments are basically considered unnecessary [18]. If patients do have symptoms, however, a variety of therapy options may be administered: foot orthoses, ankle foot orthoses, stabilising tape, exercise (training therapy with, for example, eccentric exercise), pain medication and/or anti-inflammatory medication and patient education [11, 12, 15].

Optimisation of foot loading management by means of foot orthoses and adequate footwear is the most important aspect in therapy. Depending on the progression of the pathology, this can be progressively managed with over-the-counter non-individualised foot orthoses, then with individualised foot orthoses and finally with semi-rigid ankle foot orthoses [19]. These recommendations are published in clinical guidelines, but few high-quality studies have investigated their efficacy [11, 12].

Poor quality studies with a variety of uncontrolled interventions (foot orthoses, ankle foot orthoses, strengthening exercises, custom footwear, medication, ice, mega pulse and ultrasound – isolated or combinations) report possible benefits, but the lack of high quality, robust evidence does not allow conclusions for clinical practice [1, 20–22]. The only high-quality randomised controlled trial in patients with PTTD (and pes planovalgus) found that patients benefit most from a combination of foot orthoses and exercise after a 12-week intervention period [15].

Aside from clinical outcomes, functional impairments and their possible changes through interventions are not described in the literature on the basis of biomechanical measurements. Most often, static alignment measures of foot structure like ‘navicular drop’ as a composite for excessive pronation and/or longitudinal arch flattening are used to quantify flatfoot alignment [23, 24].

However, dynamic measurement is more valid because dynamic foot motion is not strongly associated with static alignment [23, 24]. Studies on foot kinematics between flat-arched feet and controls shows that foot function and arch flattening can be quantified with multi-segment foot models during weightbearing [25, 26]. Therefore, assessing foot structure during dynamic movement seems to be the current consensus [26–28].

Evaluations of neuromuscular function are very rare. A study investigating neuromuscular activity of the tibialis posterior muscle reports enhanced activity in flat-arched asymptomatic people during stance [29]. Tibialis posterior and peroneus longus muscle activity with the use of foot orthoses in flat-arched people changed [30], which is in contrast to O’Connor et al. who report that the tibialis posterior showed (at least in running motion) inconclusive activation patterns and no changes between different shoe conditions [31]. Another study showed reduced peroneus, lateral and medial gastrocnemius, and soleus muscle activity during early stance but enhanced tibialis anterior muscle activity in participants with asymptomatic flatfoot but with a history of musculoskeletal pain compared to controls [32]. This underlines the importance of readily available neuromuscular parameters describing gait function [33–35].

Some recent studies present sophisticated measurement approaches of dynamic foot function in pes planovalgus but to the authors’ knowledge, no study to date has evaluated dynamic foot function in patients suffering from acute complaints. Most studies use healthy controls or asymptomatic flatfooted subjects [36, 23, 37]. This makes it difficult to draw clear conclusions with regard to patients.

The aims of this study are therefore to: (i) analyse the efficacy of three different non-surgical therapy regimens in PTTD patients, and (ii) evaluate descriptively functional outcome with a comprehensive biomechanical measurement approach to learn more about functional changes in movement generation and its possible plasticity throughout the intervention.

## Methods

### Study design

**Fig. 1:**
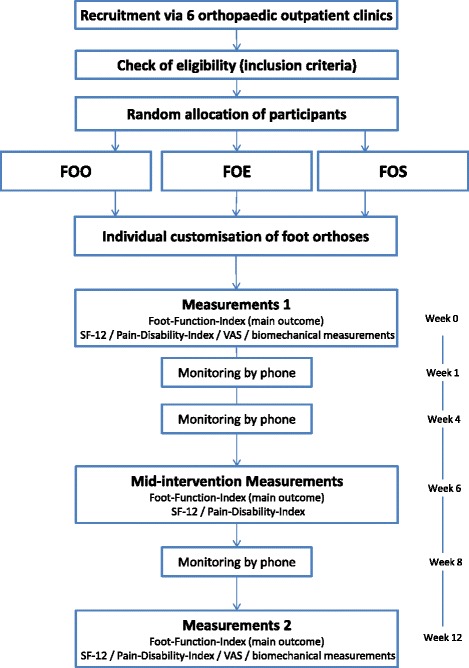
Study flow chart: randomisation of patients to one of three intervention groups: foot orthoses only FOO, foot orthoses and eccentric exercise FOE, sham foot orthoses only FOS

The study design is a parallel-group randomised trial with three intervention groups receiving therapy regimens aimed at symptoms associated with pes planovalgus (Fig. 1). There will be an intervention phase of 12 weeks, which is a common therapy window for the associated symptoms of clinical problems like pes planovalgus [38]. Participants’ pain and functional impairment perception will be measured pre- and post-intervention (and in the middle of the intervention period). Biomechanical measurements (standardised laboratory study) before and after the intervention phase will accompany the protocol to evaluate possible functional changes induced by the therapy regimen.

### Study population

A cohort of 60 participants will be recruited out of six state-wide foot surgery centres. To be included in the trial, participants will have to have: pes planovalgus, accompanying PTTD, associated foot pain; and a flexible foot structure, leading to eligibility for non-surgical treatment. Severe cases of flatfoot deformity indicating surgical correction (e.g., rigid foot structure and not being able to complete the test protocol due to severe pain or impaired physical function) will be excluded.

Specific inclusion criteria are similar to Kulig [15] and include:

- Age: 40–70 years
- Current complaint of foot and ankle pain that has lasted for three months or more
- Flexible pes planovalgus deformity in the clinical assessment
- PTTD of stage I and II according to Johnson & Strom [2]
- Pes planovalgus foot deformity with longitudinal arch flattening verified by radiograph [39]: Lateral view: lateral talo-first metatarsal angle ≠ 0° (break of axis): angle >10° according to Younger et al. [39], Anteroposterior view: anteroposterior talo-first metatarsal angle ≠ 0° (break of axis): angle >10° according to Younger et al. [39]
- No indication/not yet an indication for surgical treatment of foot deformity

Exclusion criteria are again similar to Kulig [38] and include:

- Rigid foot deformity
- PTTD of stage III and IV according to Johnson & Strom [2]
- Cardiac, neurological, peripheral vascular, or musculoskeletal pathology
- Acute infection or alcohol addiction limiting participation in study protocol
- Acute use of local or systemic analgesics
- Acute physical therapy, training therapy or physiotherapy
- Acute overuse or traumatic injury to the lower leg (excluding pes planovalgus associated pathology)
- Prior surgery to the lower limb

Participants will be included after clarification of inclusion and exclusion criteria by a clinical examination and after comprehensive written and oral information. All participants will have to sign a written informed consent in line with the Ethics Approval obtained from the Ethics Commission of the Canton Berne (Approval number: KEK-Nr. 158/12). Anthropometric data (gender, age, height, weight, shoe size and foot length) will be recorded according to guidelines of ‘Good Clinical Practice’ in subject specific Case Report Forms [40]. All subsequent data acquisition will also be documented in the Case Report Forms. This secures quality control of stored data and ensures that every single data item can be tracked down to its origin.

### Randomisation

Randomisation for group allocation to Foot Orthoses Only (FOO), Foot Orthoses with Eccentrics (FOE) or Sham Foot Orthoses (FOS) will take place with the online tool www.randomization.com (no blocking or stratification will be used). The biomechanical testing (standing, walking and walking down a stairway) will be administered in a fixed sequence since standing posture serves as calibration for dynamic measurements. Group allocation will be conducted using opaque sealed envelopes (i.e., concealed allocation). The allocation list will be stored separately and measurement personnel and data analysing personnel will not be involved in group allocation. Medical doctors, responsible for recruitment and checking of eligibility, will also not be involved in group assignment.

### Blinding

Blinding of the intervention to the participants is not possible as participants will recognise the allocated therapy. The study personnel will refrain from explaining hypothetical effects of foot orthoses, eccentric exercise or sham foot orthoses. The measurement and data analysis personnel will be blinded to group allocation during data acquisition and data analysis. De-blinding will take place after statistical analysis has been completed.

### Procedure

After the clinical examination, participants will be forwarded to the study centre for further anamnestic (medical history) pre-tests. A 1st measurement appointment will then be finalised. Meanwhile, fabrication of the foot orthoses takes place. At the 1st measurement appointment, participants’ anthropometrics and static unloaded and loaded foot posture for characterisation will be assessed. Following this, the participants’ foot health status (using the Function Index FFI, German Version (or FFI-D) [41, 42]), functional impairment (using the Pain-Disability-Index PDI [43, 44]), and current pain perception (using a VAS [38]) will be assessed. Biomechanical measurements of standing posture, level walking and walking down stairs will then be taken. After biomechanical testing, a re-evaluation of pain assessment with a VAS will be conducted.

After the 1st measurement appointment, an intervention period will follow for 12 weeks. All participants will receive a guideline for load management during the intervention and an activity diary with accompanying assessments (FFI, PDI and VAS after 6 weeks). After the 12-week intervention period, a 2nd assessment of the patient’s foot health status (FFI), functional impairment (PDI) and current pain perception (VAS pre/post-test), as well as the biomechanical tests will follow. If symptoms persist at the end of this period, participants will be transferred to the initial foot surgery centre for further medical assessment and possible ongoing therapeutic care.

### Interventions

After group allocation, all participants will be provided with instructions about their allocated interventions (see below). All participants will receive an educational session where they will be informed about the length of time to resolution of symptoms (realistic anticipation of therapy success), importance of adhering to wearing the foot orthoses, and issues about weight control [38]. A guideline based on the EdUReP (Education, Unloading, Reloading, Prevention) model, which provides a framework for management of tendinopathy, accompanies the intervention phase [45].

Participants will receive one of the following therapies according to their random allocation:

#### Foot Orthoses Only (FOO)

Participants in this group will receive custom-made foot orthoses fabricated by the same orthopaedic technician (AR, Ortho-Team AG, Bern, Switzerland). Foot orthoses will be fabricated out of Ethylene Vinyl Acetate (EVA, shore 40) with a thickness at the base of approximately 4 mm. The foot will be cast in a three-dimensional foam box impression in the neutral position of the foot under partial weightbearing [46]. This neutral position is already a “corrected” position for patients with the mentioned pathology. The 3D foam box impression serves as the basis for implementing correcting elements in the foot orthoses via computer-aided design software (CAD). A medial longitudinal arch is provided for arch support and a bowl-shaped heel is integrated to promote rear foot stability. Finally, the foot orthoses will be fabricated out of an EVA block by means of computerised numerical control technology (CNC). A dynamic barefoot plantar pressure distribution to evaluate dynamic foot function during walking (foot progression angle, forefoot abduction and medial loading) and qualitative gait analysis will be performed to support decision making on the amount of medial arch posting [43]. A medial longitudinal arch support will increase pressure at the medial plantar surface [47, 48], which might lead to changes in afferent input triggering neuromuscular control [34]. A top layer of Alcantara will be glued on top of each orthosis (Table 1). All participants will wear the device throughout the 12-week intervention for a minimum of 80 % of weight-bearing tasks.

#### Foot Orthoses and Eccentrics (FOE)

In addition to the use of the foot orthoses described above, eccentric strengthening exercises will be prescribed in this group (concentric portions are inherent, but emphasis is put on eccentric phases). Heel lowering movements will be performed on the edge of a stair from a calf raised position. For correction of rearfoot eversion a pair of socks will be squeezed between the calcaneii below the medial malleoli (active correction of rearfoot eversion and medial longitudinal arch flattening). This exercise will be performed twice per day with three sets of 15 repetitions per day for 12 weeks. Calf raises will be performed without the squeeze of the pair of socks to reduce load in the concentric phase. The exercise is a modified eccentric version of that proposed by Yuill and MacIntire [49]. It is combined with the loading suggestions of Alfredson for the treatment of Achilles tendinopathy [50, 51]. Increasing load (e.g., additional load with back pack) will be performed throughout the intervention as suggested by Jonsson [52]. Participants will be instructed by an experienced physiotherapist. They will receive an activity booklet to record all training sessions and possible accompanying activities. All participants will wear the foot orthoses throughout the 12-week intervention for a minimum of 80 % of weight-bearing tasks.

#### Foot Orthoses Sham (FOS)

This group will receive individually customised (sham) foot orthoses out of the same material described above. The functional elements of the sham orthoses, like arch support or a bowl-shaped heel to promote rearfoot stability, are designed to assist with blinding of patients. The foot will also be cast in a three-dimensional foam box impression without “correcting” to a neutral position of the foot under partial weightbearing [46]. The sham orthoses will not receive correcting elements via computer-aided design software (CAD). The sham foot orthoses will also fabricated out of an EVA block by means of computerised numerical control (CNC) technology. Like the other orthoses, a layer out of Alcantara will be glued to the top of the sham orthoses. All participants will wear the device throughout the 12-week intervention for a minimum of 80 % of weight-bearing tasks. The participants in this group will also get an activity booklet to record possible accompanying activity.

All three groups will receive a phone call after one, four and eight weeks to check for compliance, uncertainties and questions (Fig. 1). If necessary, monitoring sessions for members of the FOE group with the physiotherapist will be organised every four weeks to control exercise execution and to implement training load adjustments [52]. Where participants experience obvious fitting problems, orthoses will be slightly modified as necessary to ensure comfort and shoe fit.

### Outcome measures

#### Foot-Function-Index (FFI)

The evaluation of therapy efficacy will be conducted with the main outcome measure, the FFI, before and after the intervention. It will be administered before the biomechanical testing on the pre/post measurements. The FFI is recognised as a valid and reliable tool [53, 54]. It is also available and valid for German language use [41]. Furthermore, it has been used already to evaluate PTTD patients with pes planovalgus associated complaints and is considered to be an excellent tool in this circumstance [14, 15]. The overall (i.e., total) FFI score will be analysed as the primary outcome measure. The subscales of the FFI in the dimensions pain, disability and activity limitation will be analysed separately as secondary outcome measures.

#### Pain-Disability-Index (PDI)

The Pain-Disability-Index (PDI), used to evaluate functional impairment, will also be administered pre- and post-intervention as a secondary outcome [43, 55]. The PDI is a seven-item inventory designed to measure the degree to which pain interferes with function across a range of seven daily life dimensions (family/home responsibilities, recreation, social activity, occupation, sexual behaviour, self-care and life support activity). Each item ranges from 0 (no interference) to 10 (total interference) resulting in a sum score of 0 to 70 [56, 57, 44]. It is considered reliable for the assessment of functional impairment and chronic diseases especially in musculoskeletal pathology [58, 59]. The validity in overuse injuries has previously been proven [43, 55].

#### Visual Analogue Scale (VAS)

A 100 mm visual analogue scale (VAS) with the endpoints “no pain” and “worst pain possible” is administered before and after the biomechanical assessment to evaluate acute pain levels. It is valid and reliable in terms of the measurement of self-reported pain intensity [60].

#### Biomechanical testing

Biomechanical testing will take place before and after the intervention. All measurements will be taken with participants barefoot in three situations (“standing”, “level walking” and “walking downstairs”). Participants will walk on an even walkway with embedded force plates (AMTI® OR6, Watertown, USA) for ground contact detection and ground reaction force measurements at a self-selected speed. Ten successful walking trials will be collected. In addition, 10 successful trials of descending from two stair steps on the embedded force plates will be collected. This functionally relevant situation is administered to enhance load on the foot structures in a functionally relevant way [61, 62].

Participants will be prepared with reflective markers at bony landmarks according to a lower body marker set (Vicon® Plug-In-Gait lower body, Oxford, UK) to capture the kinematics and kinetics of hip, knee and ankle joints. A second 4-marker set based on suggestions by Dicharry will be used to measure dynamic navicular drop [23]. A static measurement will serve as a calibration for preceding dynamic trials. The comparison with the static trial will allow calculation of changes due to the additional load application during walking and on the stairway. A 10 camera setup (Vicon Bonita, Vicon® Motion Systems Ltd., Oxford, UK) will be used with a sampling rate of 200 Hz.

At the same time, the tibialis anterior, peroneal and gastrocnemius (medial and lateral head) muscles will be prepared bilaterally for surface EMG measurements. Localisation of disposable pregelled Al/AgCl bipolar surface electrodes (Ambu®, Medicotest®, DK, type N-00-S, distance from centre to centre: 25 mm) will be carefully determined according to Winter and Yack and the SENIAM procedures [63, 64]: The longitudinal axes of the electrodes shall be in line with the presumed direction of the underlying muscle fibres. Interelectrode impedance shall be kept below 5 kOhms by means of shaving, light abrasion, degreasing and disinfecting the skin with alcohol. The EMG electrodes will be directly connected to differential pre-amplifiers and taped to the skin. The pre-amplified signals will then be transmitted via shielded cables (fixed to the leg with Velcro straps lattice bracing) to the main amplifier (PowerPack, pfitec®, Endingen, DE). Signals will be 12-bit analogue/digital-converted and stored on a personal computer. The sampling frequency will be set at 2000 Hz.

Signal data processing of kinematic data will calculate a representative mean stride cycle from the 10 collected trials per situation and condition. The kinematic outcome measures will be calculated with the manufacturer’s software Vicon Nexus® (Vicon® Motion Systems Ltd., Oxford, UK), and MatLab® (Mathworks Inc., Natick, USA) and will then be transferred to a data spreadsheet for statistical analysis.

Post-processing of EMG signals will include rectification and calculation of one average stride cycle out of 10 consecutive stride cycles. The on-off pattern of muscle activity will then be defined above a threshold of the resting signal plus two standard deviations. Above this threshold the muscle will be considered “on”. “Off” is defined after decline of the EMG signal below this threshold. “On” and “off” will be determined automatically by customised software (LabView®-based: Software Imago, pfitec®, Endingen, DE) and checked visually by a person experienced in EMG data processing but blinded to group assignment of the individual trial. This is considered to be a reasonable procedure securing both high standardisation and validity [65]. Total time of activation (Ttot) will then be calculated as a percentage of total stride time. The onset of activation (Tini) and the time of maximum activation (Tmax) will be extracted and expressed in relation to touchdown in % of stride [66, 34, 33, 35]. Amplitudes in the gait cycle phases pre-activation (Apre), weight acceptance (Awa), midstance (Ams) and push-off (Apo) will be calculated according to the phase definition by Winter [67]: Pre-activation is defined as the period from onset of electromyographic activity (see above) to initial touchdown. Weight acceptance is the period of time between initial contact and the following 15 % of the stride period (‘early stance’). Midstance is the period from 15 % to 40 % of stride. Push-off is the period of time late in stance from 40 % of stride to toe-off [67]. The mean amplitude voltage (MAV) per gait cycle phase will be extracted and normalised to the MAV of the entire stride cycle. Hence, Apre, Awa, Ams and Apo are determined relative to the average activity and expressed as a fraction of the average amplitude (=1.0). Data will then be transferred from LabVIEW®-format to a data spreadsheet for plausibility control and statistical analysis.

Signal processing personnel will be blinded to experimental condition. The electronic database will then be checked for plausibility. All measures will be checked by range checks to estimate validity of retrieved quantities. Apparent discordant values will be tracked back to their origin and data analysis will be repeated. The data spreadsheet of all manually collected data like anthropometrics will be printed as a hardcopy. Missing values will be marked, cross-checked, added or corrected if applicable. Random re-evaluation of original data will serve for cross-checking of originally analysed biomechanical data. All outcomes and quantities of kinematic and electromyographic measurements are shown in Table 1 (primary, secondary and tertiary outcome measures).

**Table 1:** Outcome measures

Primary outcome measure	Unit
• Foot Function Index (FFI) – total score	sum score [0–230]
Secondary outcome measures	Unit
• Foot Function Index (FFI) – subcategory pain	sum score [0–90]
• Foot Function Index (FFI) – subcategory disability	sum score [0–90]
• Foot Function Index (FFI) – subcategory activity limitation	sum score [0–50]
Tertiary outcome measures	Unit
Additional assessment of functional impairment and current pain perception	
• Pain Disability Index	Sum score [0–70]
• Visual Analog Scale (VAS) assessing pain (pre-post biomechanical testing)	Score [0–10]
Tertiary outcome measures from biomechanical data	Unit
Kinematic data from 3D movement analysis	
Distance	
• Dynamic navicular drop	Millimeter [mm]
Kinematic data from 3D movement analysis	
Angular data: angle at initial contact, maximal manifestation during stance, range	
• Foot Progression Angle	Angular degree [°]
• Forefoot to rearfoot dorsiflexion	Angular degree [°]
• Forefoot to rearfoot adduction	Angular degree [°]
• Forefoot to rearfoot supination	Angular degree [°]
• Ankle dorsiflexion	Angular degree [°]
• Ankle adduction	Angular degree [°]
• Ankle eversion	Angular degree [°]
(hindfoot with respect to tibia)	
• Ankle dorsiflexion	Angular degree [°]
• Ankle adduction	Angular degree [°]
• Ankle eversion	Angular degree [°]
(hindfoot with respect to lab)	
• Knee flexion	Angular degree [°]
• Knee adduction	Angular degree [°]
• Knee internal rotation	Angular degree [°]
• Hip flexion	Angular degree [°]
• Hip adduction	Angular degree [°]
• Hip internal rotation	Angular degree [°]
Neuromuscular activity	
EMG of M. tibialis anterior, M. peroneus longus, M. gastrocnemius lateralis/medialis, M. soleus	
• Onset of activation	% of stride
• Time of maximum activation	% of stride
• Total time of activation	% of stride
• Normalized amplitude in preactivation	arbitrary unit [uV/uV]
• Normalized amplitude in weight acceptance	arbitrary unit [uV/uV]
• Normalized amplitude in mid-stance	arbitrary unit [uV/uV]
• Normalized amplitude in push-off	arbitrary unit [uV/uV]

Primary, secondary and tertiary outcome measures of this RCT

### Data analysis

Data will be transferred from Case Report Forms to Microsoft® Excel for Windows (current version) spread sheets and later processed by SPSS® (current version). Biomechanical data will be processed with the outlined software and final quantities will also be transferred to spreadsheets for statistical analysis.

Statistical analysis will be performed descriptively after plausibility control. First, testing of normal distribution (Shapiro-Wilk test) of data will be followed by the calculation of mean, standard deviation and 95 % confidence interval. Primary outcome measure (FFI total score) at measurement points 0, week 6 and week 12 will be analysed by 3x3 Analysis of Variance (ANOVA) with repeated measures identifying differences in the FFI across groups (FOO, FOE, FOS) and test sessions. This also applies for the secondary outcome measures (FFI pain subcategory, FFI disability subcategory and FFI activity limitation subcategory). The primary endpoint will be set at 12 weeks. Biomechanical data will be analysed descriptively. Pre-post comparisons of those tertiary outcome measures will also be performed where indicated after descriptive analysis by 2×2 repeated measures ANOVA (factor group and test day).

Sample size calculation has been based on the outlined protocol with three intervention groups and the same primary outcome as Kulig et al. [14, 15], where a sample size of *n* = 15 per group (total *N* = 45) was determined based on clinical observation, attrition rate and power analysis. Clinical observation suggested that there is moderate to high inter-subject variability with respect to pain, disability and function (patient’s perception). A drop-out rate of 20 % is assumed due to a long intervention period, increase or resolution of symptoms during the intervention, or a need for surgery requiring exit from the study. Power analysis was based on an alpha level of 0.05 and beta of 0.8 resulting in the proposed sample size of 45 [38].

Based on this reported sample size with the same main outcome and design, an *à priori* calculation of the total sample size was performed leading to a group sample of *n* = 20 (total *N* = 60) according to the following assumptions (Software: G*Power3, [68, 69]: test family = F-tests, statistical test = repeated measures ANOVA, effect size = 0.2 (medium), alpha = 0.05, power = 0.82, number of groups = 3, number of measurements = 3, correlation among the repeated measures = 0.5, nonsphericity correction epsilon = 1 and dropouts *n* = 6).

## Discussion

The objective of this randomised trial is the evaluation of the therapeutic benefit of three different non-surgical treatment regimens (foot orthoses only FOO, foot orthoses and eccentric exercise FOE, sham foot orthoses FOS) in patients with pes planovalgus and accompanying complaints. The clinical validity of those therapy regimens will result in important clinical implications.

The lack of evidence for non-surgical treatment strategies emphasises the need for such a trial. Beside the fact that established clinical outcomes will be used to assess clinical efficacy of therapy regimens, the novel aspect of the trial is to integrate biomechanical measurements of lower extremity and foot function.

The development and final set-up of the intervention groups was the subject of great debate between the investigators, the funding agency’s international reviewers and the ethics committee. The author/investigator group initially planned foot orthoses as the basic therapy for all three groups. The promising results of the aforementioned RCT by Kulig et al. [15] justified this approach [38]. Consequently, according to the best available evidence, foot orthoses were planned for all groups. One group with additional eccentric exercises and one group with additional “traditional” physiotherapy (a regimen of ice, ultrasound, electrotherapy, manual therapy) were planned to assess the additive effect of exercise therapy and physiotherapy. The rationale for eccentric exercises was derived from existing evidence for the treatment of (chronic) tendinopathy [51, 50, 52]. Physiotherapy was planned to be integrated into this trial because it reflects current physiotherapeutic practice in the daily routine. The reviewers of the funding agency rejected the physiotherapy therapy modality due to lack of any scientific evidence. During the process of ethics approval, the regional ethics committee, acting as the legal authority in Switzerland came to the conclusion that there is also no rationale for foot orthoses since the trial by Kulig et al. did not evaluate foot orthoses against a wait-and-see approach [15]. They concluded that research ethics allow refraining from foot orthoses in this patient group. Consequently, they requested an untreated control group. The investigator group emphasised that there are frequent dropouts in such a control group and suggested a sham orthosis group as a control. The ethics committee finally approved this idea and the funding agency also accepted the final therapy group definitions.

The rationale for the measurement of the gait mechanics and neuromuscular control in this trial is to gain a comprehensive view on functional changes due to pathology and their plasticity during the intervention. One study has analysed kinematic differences between healthy and PTTD patients in a heel rise movement [70]. It found that stage II PTTD patients exhibited greater plantar flexion to achieve heel rise compared to healthy controls [70]. The same study found greater tibialis posterior muscle lengthening during walking and greater hindfoot eversion during stance [71, 72].

The tibialis posterior muscle is the main muscular stabiliser for active longitudinal arch stabilisation [73]. Foot orthoses with an arch support have been found to selectively activate the tibialis posterior muscle [74]. Therefore, it would be the best muscular indicator in the context of pes planovalgus, but measurements of this deep lower leg muscle are not possible with surface electromyography. Accordingly, the trial refrains from using highly invasive techniques, so as to not place study participants at risk. There are reports of synergistic activity of the tibialis posterior muscle and the superficially available peroneus longus muscle [29]. The approach is, therefore, to evaluate easily available parameters that can be transferred to clinical settings. Further assessment of the tibialis anterior muscle and gastrocnemius (medialis and lateralis) muscles [66, 34, 33, 35] will be performed to cover the “neuromuscular” dimension that relate to common kinematic movements like dynamic navicular drop, rearfoot eversion, etc.

The analysis of possible functional changes in gait mechanics and neuromuscular control will contribute to an enhanced understanding of functional changes that occur with conservative management. This will enable us to generate theories about the mechanisms possibly involved in the process of symptom relief. Furthermore, a subsequent aim is to optimise conservative management strategies, which may lead to more efficient use of health care resources.

## References

[1] Chao W, Wapner KL, Lee TH, Adams J, Hecht PJ (1996). Nonoperative management of posterior tibial tendon dysfunction. Foot Ankle Int.

[2] Johnson KA, Strom DE (1989). Tibialis posterior tendon dysfunction. Clin Orthop Relat Res.

[3] Mann RA (1983). Acquired flatfoot in adults. Clin Orthop Relat Res.

[4] Rao UB, Joseph B (1992). The influence of footwear on the prevalence of flat foot. A survey of 2300 children. J Bone Joint Surg (Br).

[5] Vittore D, Patella V, Petrera M, Caizzi G, Ranieri M, Putignano P (2009). Extensor deficiency: first cause of childhood flexible flat foot. Orthopedics.

[6] Pritsch T, Maman E, Steinberg E, Luger E (2004). Posterior tibial tendon dysfunction. Harefuah.

[7] Imhauser CW, Siegler S, Abidi NA, Frankel DZ (2004). The effect of posterior tibialis tendon dysfunction on the plantar pressure characteristics and the kinematics of the arch and the hindfoot. Clin Biomech (Bristol, Avon).

[8] Yeap JS, Singh D, Birch R (2001). Tibialis posterior tendon dysfunction: a primary or secondary problem?. Foot Ankle Int.

[9] Pomeroy GC, Pike RH, Beals TC, Manoli A (1999). Acquired flatfoot in adults due to dysfunction of the posterior tibial tendon. J Bone Joint Surg Am.

[10] Richie DH (2005). Pathomechanics of the adult acquired flatfoot. Foot Ankle Quart.

[11] Bowring B, Chockalingam N (2009). A clinical guideline for the conservative management of tibialis posterior tendon dysfunction. Foot (Edinb).

[12] Bowring B, Chockalingam N (2010). Conservative treatment of tibialis posterior tendon dysfunction--a review. Foot (Edinb).

[13] Kohls-Gatzoulis J, Angel JC, Singh D, Haddad F, Livingstone J, Berry G (2004). Tibialis posterior dysfunction: a common and treatable cause of adult acquired flatfoot. Br Med J.

[14] Kulig K, Lederhaus ES, Reischl S, Arya S, Bashford G (2009). Effect of eccentric exercise program for early tibialis posterior tendinopathy. Foot Ankle Int.

[15] Kulig K, Reischl SF, Pomrantz AB, Burnfield JM, Mais-Requejo S, Thordarson DB (2009). Nonsurgical management of posterior tibial tendon dysfunction with orthoses and resistive exercise: a randomized controlled trial. Phys Ther.

[16] Bluman EM, Title CI, Myerson MS (2007). Posterior tibial tendon rupture: a refined classification system. Foot Ankle Clin.

[17] Gluck GS, Heckman DS, Parekh SG (2010). Tendon disorders of the foot and ankle, part 3: the posterior tibial tendon. Am J Sports Med.

[18] Pfeiffer M, Kotz R, Ledl T, Hauser G, Sluga M (2006). Prevalence of flat foot in preschool-aged children. Pediatrics.

[19] Wapner KL, Chao W (1999). Nonoperative treatment of posterior tibial tendon dysfunction. Clin Orthop Relat Res.

[20] Alvarez RG, Marini A, Schmitt C, Saltzman CL (2006). Stage I and II posterior tibial tendon dysfunction treated by a structured nonoperative management protocol: an orthosis and exercise program. Foot Ankle Int.

[21] Bek N, Oznur A, Kavlak Y, Uygur F (2003). The effect of orthotic treatment of posterior tibial tendon insufficiency on pain and disability. Pain Clin.

[22] Jari S, Roberts N, Barrie J (2002). Non-surgical management of tibialis posterior insufficiency. Foot Ankle Surg.

[23] Dicharry JM, Franz JR, Della Croce U, Wilder RP, Riley PO, Kerrigan DC (2009). Differences in static and dynamic measures in evaluation of talonavicular mobility in gait. J Orthop Sports Phys Ther.

[24] Hargrave MD, Carcia CR, Gansneder BM, Shultz SJ (2003). Subtalar pronation does not influence impact forces or rate of loading during a single-leg landing. J Athl Train.

[25] Levinger P, Murley GS, Barton CJ, Cotchett MP, McSweeney SR, Menz HB (2010). A comparison of foot kinematics in people with normal- and flat-arched feet using the Oxford foot model. Gait Posture.

[26] Nielsen RG, Rathleff MS, Moelgaard CM, Simonsen O, Kaalund S, Olesen CG (2010). Video based analysis of dynamic midfoot function and its relationship with foot posture index scores. Gait Posture.

[27] Leung AK, Cheng JC, Zhang M, Fan Y, Dong X (2004). Contact force ratio: a new parameter to assess foot arch function. Prosthet Orthot Int.

[28] Shanthikumar S, Low Z, Falvey E, McCrory P, Franklyn-Miller A (2010). The effect of gait velocity on calcaneal balance at heel strike; implications for orthotic prescription in injury prevention. Gait Posture.

[29] Murley GS, Buldt AK, Trump PJ, Wickham JB (2009). Tibialis posterior EMG activity during barefoot walking in people with neutral foot posture. J Electromyogr Kinesiol.

[30] Murley GS, Landorf KB, Menz HB (2010). Do foot orthoses change lower limb muscle activity in flat-arched feet towards a pattern observed in normal-arched feet?. Clin Biomech (Bristol, Avon).

[31] O’Connor KM, Hamill J (2004). The role of selected extrinsic foot muscles during running. Clin Biomech (Bristol, Avon).

[32] Hunt AE, Smith RM (2004). Mechanics and control of the flat versus normal foot during the stance phase of walking. Clin Biomech (Bristol, Avon).

[33] Baur H, Leuenberger C (2011). Analysis of ratios in multivariate morphometry. Syst Biol.

[34] Baur H, Hirschmuller A, Muller S, Mayer F (2011). Neuromuscular activity of the peroneal muscle after foot orthoses therapy in runners. Med Sci Sports Exerc.

[35] Baur H, Muller S, Hirschmuller A, Cassel M, Weber J, Mayer F (2011). Comparison in lower leg neuromuscular activity between runners with unilateral mid-portion Achilles tendinopathy and healthy individuals. J Electromyogr Kinesiol.

[36] Bandholm T, Boysen L, Haugaard S, Zebis MK, Bencke J (2008). Foot medial longitudinal-arch deformation during quiet standing and gait in subjects with medial tibial stress syndrome. J Foot Ankle Surg.

[37] Nielsen RG, Rathleff MS, Simonsen OH, Langberg H (2009). Determination of normal values for navicular drop during walking: a new model correcting for foot length and gender. J Foot Ankle Res.

[38] Kulig K, Pomrantz AB, Burnfield JM, Reischl SF, Mais-Requejo S, Thordarson DB (2006). Non-operative management of posterior tibialis tendon dysfunction: design of a randomized clinical trial [NCT00279630]. BMC Musculoskelet Disord.

[39] Younger AS, Sawatzky B, Dryden P (2005). Radiographic assessment of adult flatfoot. Foot Ankle Int.

[40] ICH. International conference on harmonisation of technical requirements for registration of pharmaceuticals for human use: ICH harmonised tripartite guideline. Guideline for good clinical practice E6(R1). 1996. http://www.ich.org/fileadmin/Public_Web_Site/ICH_Products/Guidelines/Efficacy/E6/E6_R1_Guideline.pdf. Accessed 1 July 2015.

[41] Naal FD, Impellizzeri FM, Huber M, Rippstein PF (2008). Cross-cultural adaptation and validation of the Foot Function Index for use in German-speaking patients with foot complaints. Foot Ankle Int.

[42] Budiman-Mak E, Conrad KJ, Mazza J, Stuck RM (2013). A review of the foot function index and the foot function index - revised. J Foot Ankle Res.

[43] Hirschmüller A, Baur H, Müller S, Helwig P, Dickhuth HH, Mayer F (2011). Clinical effectiveness of customised sport shoe orthoses for overuse injuries in runners: a randomised controlled study. Br J Sports Med.

[44] Tait RC, Chibnall JT, Krause S (1990). The pain disability index: psychometric properties. Pain.

[45] Davenport TE, Kulig K, Matharu Y, Blanco CE (2005). The EdUReP model for nonsurgical management of tendinopathy. Phys Ther.

[46] Ki SW, Leung AK, Li AN (2008). Comparison of plantar pressure distribution patterns between foot orthoses provided by the CAD-CAM and foam impression methods. Prosthet Orthot Int.

[47] Baur H, Hirschmüller A, Müller S, Mayer F (2003). Effects of functional elements of orthotic insoles in sports. Deut Z Sportmed.

[48] Redmond AC, Landorf KB, Keenan AM (2009). Contoured, prefabricated foot orthoses demonstrate comparable mechanical properties to contoured, customised foot orthoses: a plantar pressure study. J Foot Ankle Res.

[49] Yuill EA, Macintyre IG (2010). Posterior tibialis tendonopathy in an adolescent soccer player: a case report. J Can Chiropr Assoc.

[50] Alfredson H (2003). Chronic midportion Achilles tendinopathy: an update on research and treatment. Clin Sports Med.

[51] Alfredson H, Pietila T, Jonsson P, Lorentzon R (1998). Heavy-load eccentric calf muscle training for the treatment of chronic Achilles tendinosis. Am J Sports Med.

[52] Jonnson P, Alfredson D, Sunding K, Fahlstrom M, Cook J (2008). New regimen for eccentric calf-muscle training in patients with chronic insertional Achilles tendinopathy: results of a pilot study. Br J Sports Med.

[53] Budiman-Mak E, Conrad KJ, Roach KE (1991). The Foot Function Index: a measure of foot pain and disability. J Clin Epidemiol.

[54] Martin RL, Irrgang JJ (2007). A survey of selfreported outcome instruments for the foot and ankle. J Orthop Sports Phys Ther.

[55] Mayer F, Hirschmuller A, Muller S, Schuberth M, Baur H (2007). Effects of short-term treatment strategies over 4 weeks in Achilles tendinopathy. Br J Sports Med.

[56] Hankin HA, Killian CB (2004). Prediction of functional outcomes in patients with chronic pain. Work.

[57] Jerome A, Gross RT (1991). Pain disability index: construct and discriminant validity. Arch Phys Med Rehabil.

[58] Gronblad M, Hupli M, Wennerstrand P, Jarvinen E, Lukinmaa A, Kouri JP (1993). Intercorrelation and test-retest reliability of the Pain Disability Index (PDI) and the Oswestry Disability Questionnaire (ODQ) and their correlation with pain intensity in low back pain patients. Clin J Pain.

[59] Rittweger J, Just K, Kautzsch K, Reeg P, Felsenberg D (2002). Treatment of chronic lower back pain with lumbar extension and whole-body vibration exercise: a randomized controlled trial. Spine.

[60] Scott J, Huskisson EC (1976). Graphic representation of pain. Pain.

[61] Leitner M, Schmid S, Hilfiker R, Radlinger L (2011). Test-retest reliability of vertical ground reaction forces during stair climbing in the elderly population. Gait Posture.

[62] Luder G, Baumann T, Jost C, Schmid S, Radlinger L (2007). Variability of Ground Reaction Forces in Healthy Subjects during Stair Climbing. Physioscience.

[63] Hermens HJ, Freriks B, Disselhorst-Klug C, Rau G (2000). Development of recommendations for SEMG sensors and sensor placement procedures. J Electromyogr Kinesiol.

[64] Winter DA, Yack HJ (1987). EMG profiles during normal human walking: stride-to-stride and inter-subject variability. Electroencephalogr Clin Neurophysiol.

[65] Hodges PW, Bui BH (1996). A comparison of computer-based methods for the determination of onset of muscle contraction using electromyography. Electroencephalogr Clin Neurophysiol.

[66] Baur H, Hirschmuller A, Cassel M, Muller S, Mayer F (2010). Gender-specific neuromuscular activity of the M. peroneus longus in healthy runners - A descriptive laboratory study. Clin Biomech (Bristol, Avon).

[67] Winter DA (1991). Biomechanics and Motor Control of Human Gait: Normal, Elderly and Pathological.

[68] Faul F, Erdfelder E, Buchner A, Lang AG (2009). Statistical power analyses using G*Power 3.1: tests for correlation and regression analyses. Behav Res Methods.

[69] Faul F, Erdfelder E, Lang AG, Buchner A (2007). G*Power 3: a flexible statistical power analysis program for the social, behavioral, and biomedical sciences. Behav Res Methods.

[70] Houck JR, Neville C, Tome J, Flemister AS (2009). Foot kinematics during a bilateral heel rise test in participants with stage II posterior tibial tendon dysfunction. J Orthop Sports Phys Ther.

[71] Houck JR, Neville CG, Tome J, Flemister AS (2009). Ankle and foot kinematics associated with stage II PTTD during stance. Foot Ankle Int.

[72] Neville C, Flemister A, Tome J, Houck J (2007). Comparison of changes in posterior tibialis muscle length between subjects with posterior tibial tendon dysfunction and healthy controls during walking. J Orthop Sports Phys Ther.

[73] Kulig K, Burnfield JM, Requejo SM, Sperry M, Terk M (2004). Selective activation of tibialis posterior: evaluation by magnetic resonance imaging. Med Sci Sports Exerc.

[74] Kulig K, Burnfield JM, Reischl S, Requejo SM, Blanco CE, Thordarson DB (2005). Effect of foot orthoses on tibialis posterior activation in persons with pes planus. Med Sci Sports Exerc.

